# Dr. Nelson D. Blood

**Published:** 1916-05

**Authors:** 


**Dr. Nelson D. Blood,** Bellevue 1874, (not listed in State Directory, given in Polk) died at his home in Auburn March 11, aged 72.

